# Global analysis of DNA methylation in young (J1) and senescent (J2) *Gossypium hirsutum* L. cotyledons by MeDIP-Seq

**DOI:** 10.1371/journal.pone.0179141

**Published:** 2017-07-17

**Authors:** Lingling Dou, Xiaoyun Jia, Hengling Wei, Shuli Fan, Hantao Wang, Yaning Guo, Shan Duan, Chaoyou Pang, Shuxun Yu

**Affiliations:** 1 State Key Laboratory of Cotton Biology, Institute of Cotton Research of CAAS, Anyang, Henan, P. R. China; 2 Weinan Institute of Agricultural Sciences, Weinan, Shaanxi, P. R. China; University of Perugia, ITALY

## Abstract

DNA methylation is an important epigenetic modification regulating gene expression, genomic imprinting, transposon silencing and chromatin structure in plants and plays an important role in leaf senescence. However, the DNA methylation pattern during *Gossypium hirsutum* L. cotyledon senescence is poorly understood. In this study, global DNA methylation patterns were compared between two cotyledon development stages, young (J1) and senescence (J2), using methylated DNA immunoprecipitation (MeDIP-Seq). Methylated cytosine occurred mostly in repeat elements, especially LTR/Gypsy in both J1 and J2. When comparing J1 against J2, there were 1222 down-methylated genes and 623 up-methylated genes. Methylated genes were significantly enriched in carbohydrate metabolism, biosynthesis of other secondary metabolites and amino acid metabolism pathways. The global DNA methylation level decreased from J1 to J2, especially in gene promoters, transcriptional termination regions and regions around CpG islands. We further investigated the expression patterns of 9 DNA methyltransferase-associated genes and 2 DNA demethyltransferase-associated genes from young to senescent cotyledons, which were down-regulated during cotyledon development. In this paper, we first reported that senescent cotton cotyledons exhibited lower DNA methylation levels, primarily due to decreased DNA methyltransferase activity and which also play important role in regulating secondary metabolite process.

## Background

DNA methylation is a critical epigenetic modification that is wide spread in plants. It maintains chromatin structure, DNA conformation, and DNA stability and alters DNA-protein interactions [1].

There are currently three approaches for DNA methylation analysis; one depends on DNA base conversion, such as bisulfite-sequencing PCR (BSP) [2]. The other approaches, using methylation-sensitive amplified polymorphism (MSAP) [3] and methylation DNA-enriched sequencing (MeDIP-Seq), are independent of DNA conversion [4]. DNA base conversion methods require extensive work, and MeDIP-Seq is a comparable cost-effective and efficient method to investigate genome-wide DNA methylation. MeDIP-seq uses a 5-methylcytosine antibody to enrich for DNA fragments containing this modification, and the enriched DNA fragments are then sequenced using high-throughput methods.

In plants, during the methylation process, the receptor acquires a methyl group from the corresponding methyl donor S-adenosylmethionine (SAM) during methyltransferase catalysis, which constitutes the primary form of 5-methylcytosine methylation receptors. DNA methylation occurs at different genetic components and has different functions regulating gene expression. In some cases, DNA methylation occurs in the promoter, the first exon and the transcriptional termination region and usually results in gene silencing [5–7]. However, understanding how modification occurring elsewhere in the gene body control expression is complex [8]. Therefore, plants could control gene expression through methylation and demethylation with temporal and spatial patterns during development.

Recent studies have reported that DNA methylation is one of the most important epigenetic modifications regulating plant senescence [9]. Zhu and co-workers [10] reported that silencing *SlELP2L* (an elongator complex protein 2-like gene) in tomatoes inhibits leaf growth and accelerates leaf and sepal senescence by increasing DNA methyltransferase gene expression. Genomic DNA methylation analysis during the reinvigoration of *Pinus radiata* indicated that DNA methylation decreased as the degree of reinvigoration increased in meristematic areas [11]. DNA methylation increased with maturation and conversely decreased with rejuvenation at the shoot tips and apical meristems in *Eucalyptus*, determined using high-performance liquid chromatography (HPLC) methods [12]. Total genomic DNA methylation rates in *Moso bamboo* were significantly different at different chronological ages, and increased genomic DNA methylation rate correlated with an increase in chronological age [13]. HPLC analyses in *Acacia mangium Willd* observed increased DNA methylation in microshoots with juvenile leaf morphology than in the mature phyllode morphology [14]. A decrease in viability during *Quercus robur* seed aging highly correlated with a global decline in the levels of 5-methylcotysine in genomic DNA. Therefore, this decrease in methylation might represent a typical response to aging and senescence in recalcitrant seeds [15]. DNA methylation increased in some plant tissues with age, whereas it decreased in others. However, the global DNA methylation patterns from young to senescent *G*. *hirsutum* L.cotyledons remain unknown.

Leaf senescence is the last stage of plant development and can be characterized by material degradation and recycling, leading to plant death and causing tissue aging due to environmental stresses or internal factors [16]. Early senescence is also known as premature senescence, and short-season cotton is usually accompanied by premature senescence [17]. Premature senescence of cotton resulted in a reduction in cotton fiber yield and quality [18]. Cotton is one of the most important economic crops and premature senescence of cotton is one of the most important restricted factors in China [19]. Therefore, it is important to study the mechanism of cotton leaf senescence.

While DNA methylation is one of the most important epigenetic modifications, there are few reports about DNA methylation in cotton [20]. Osabe et al. [21] investigated DNA methylation in various tissues from *G*. *hirsutum* L. and *G*. *barbadense* L. cotton plants and found that the differences in DNA methylation were more pronounced than genetic differences between the genotypes. Silencing of the REPRESSOR OF SILENCING 1 (ROS1) gene promoted DNA methylation and significantly repressed fiber elongation in August in *G*. *hirsutum* L. cv. Xuzhou 142 [22]. Min and co-workers speculated that increased histone methylation might compensate for the low levels of DNA methylation level in *G*. *hirsutum* L. cv. H05 (sensitive to high temperature) under high temperatures [23]. However, more work in cotton is required.

In this study, we compared DNA methylation patterns between young (J1) and senescent cotyledons (J2) by MeDIP-seq. DNA methylation-associated significantly enriched biochemical pathway analysis revealed that DNA methylation is involved in regulating carbohydrate metabolism, biosynthesis of other secondary metabolites and amino acid metabolism pathways during the cotyledon senescent process. DNA methylation levels decreased from young to senescent cotyledons, which was likely due to their decreased expression of DNA methylation-associated genes as DNA methyltransferase-associated gene expression was down-regulated from young to senescent cotyledons.

## Results

### Overview of MeDIP-Seq data in young and senescent *G*. *hirsutum* L. cotyledons

**Fig 1:**
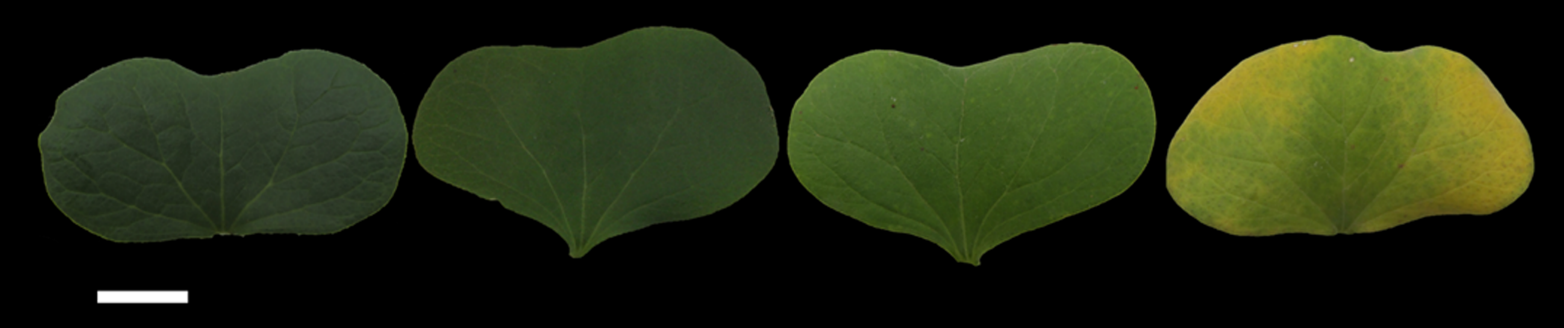
Plant phenotypes at four developmental stages.

To analyze genome-wide DNA methylation in young and senescent cotton cotyledons (Fig 1), we generated 81,632,654 reads from young cotyledon samples (J1) and senescent cotyledon samples (J2) using paired-end, 49-bp methods. A total of 95.68% and 95.65% of the total read mapped to the J1 and J2 reference genomes, respectively, of which 69.49% and 69.74% mapped to specific regions of the *G*. *hirsutum* L. genome (Table 1). Genome coverage analysis of the three 5-methylcytosine forms CG, CHG and CHH (H indicates A, T or G) sites negatively correlated with sequencing depth. A large number of regions showed low depth coverage, whereas a small proportion of regions exhibited high sequencing depth coverage, and the percentages of CpGs and CHG in J1 were higher than in J2 (S1 Table). As shown in S2 Table, most of the reads tended to cluster in regions with low numbers of CpGs, which ranged from 5 to 35 in both J1 and J2.

**Table 1:** Data generated by MeDIP-Seq analysis of young (J1) and senescent cotyledons (J2).

Sample	Total reads	Mapped reads	Mapping rate (%)	Effective Chain Depth	Unique Mapped reads	Unique Mapping Rate (%)
J1	81,632,654	78,104,228	95.68	1.78	56,724,818	69.49
J2	81,632,654	78,078,167	95.65	1.78	56,934,550	69.74

**Fig 2:**
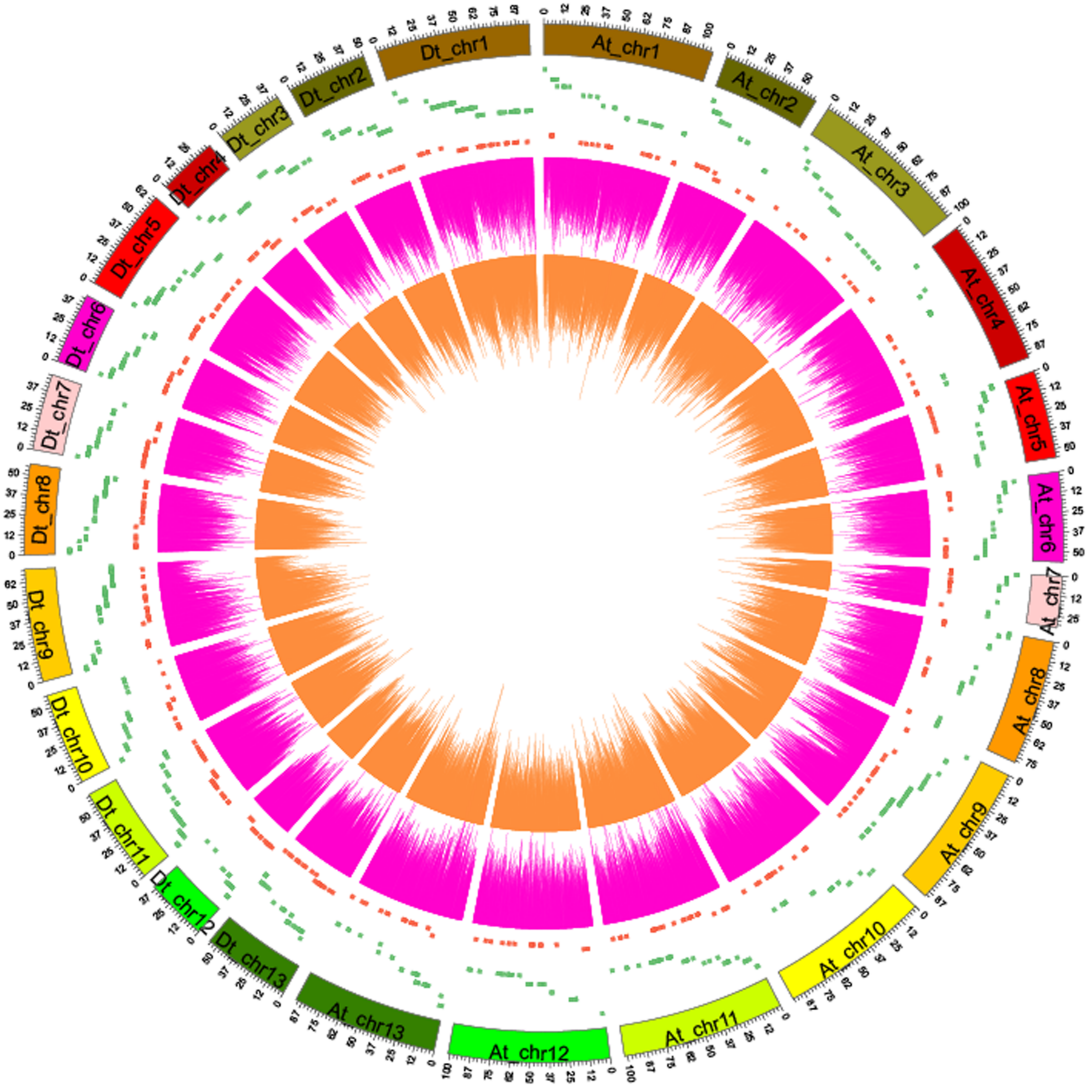
Distribution of MeDIP-Seq reads and the density of different methylated genes on each chromosome between J1 and J2. Orange lines indicate reads distributions in J1; purple lines indicate reads distribution in J2. The green dots indicate down-methylated genes in J2 compared to J1, whereas the red dots indicate up-methylated genes. At and Dt indicate subgenomes of allotetraploid *G*. *hirsutum* L,. respectively.

The MeDIP-Seq reads were distributed across all 26 chromosomes and 9127 scaffolds and were broadly spread throughout most chromosomal regions. We counted read number stepwise using 10 kb/window on each chromosome and physically visualized the reads on chromosomes with line graphs (Fig 2), which also reflects read enrichment on every chromosome without obvious preference or deficiency. Analysis of the genome coverage distribution across sequencing depth showed that sequencing depth≥1 makes up more than 44% of the genome of J1 and J2 (S3 Table).

We used uniquely mapped reads to analyze repetitive elements annotated in the *G*. *hirsutum* L. genome [24]. Repetitive elements showed very high DNA methylation proportions, with more than half of the uniquely mapped reads localizing to repetitive elements. We also found that different repeat elements had different DNA methylation levels (Table 2). LTR/Gypsy and LTR/Copia were the most widely methylated DNA repetitive elements in both J1 and J2. There were 59.75% LTR/Gypsy and 7.32% LTR/Copia in J1 and 58.72% LTR/Gypsy and 7.59% LTR/Copia in J2. According to the results, 5-methylcytosine primarily occurred in repeat elements of *G*. *hirsutum* L. cotyledons.

**Table 2:** Distribution of reads in repetitive elements in J1 and J2.

Repetitive_elements	J1	J2
LTR/Gypsy	59.75	58.72
LTR/Copia	7.32	7.59
LTR	4.83	4.74
LINE/L1	1.83	2.06
TRF	0.87	0.91
DNA/MuDR	0.82	0.81
Unknown	0.42	0.44
DNA/CMC-EnSpm	0.27	0.28
Simple_repeat	0.26	0.26
DNA/MULE-MuDR	0.25	0.24
DNA/En-Spm	0.19	0.19
DNA/hAT-Ac	0.14	0.14
DNA/Harbinger	0.12	0.13
DNA/PIF-Harbinger	0.1	0.1
DNA	0.1	0.1
LTR/Caulimovirus	0.09	0.1
DNA/hAT-Tip100	0.07	0.07
LINE/Penelope	0.07	0.08
RC/Helitron	0.05	0.05
DNA/hAT-Tag1	0.04	0.05
Satellite	0.04	0.04

However, only a small proportion of uniquely mapped MeDIP-seq reads mapped to different gene elements, including CpG islands, 2k upstream, 5’- UTR, CDS, intron, 3’- UTR, and 2k downstream (S3 Table). The uniquely mapped MeDIP-seq reads were primarily distributed in the 2k upstream and 2k downstream regions, followed by the gene body.

### Characterization of MeDIP-seq reads around the gene body and CpG islands

**Fig 3:**
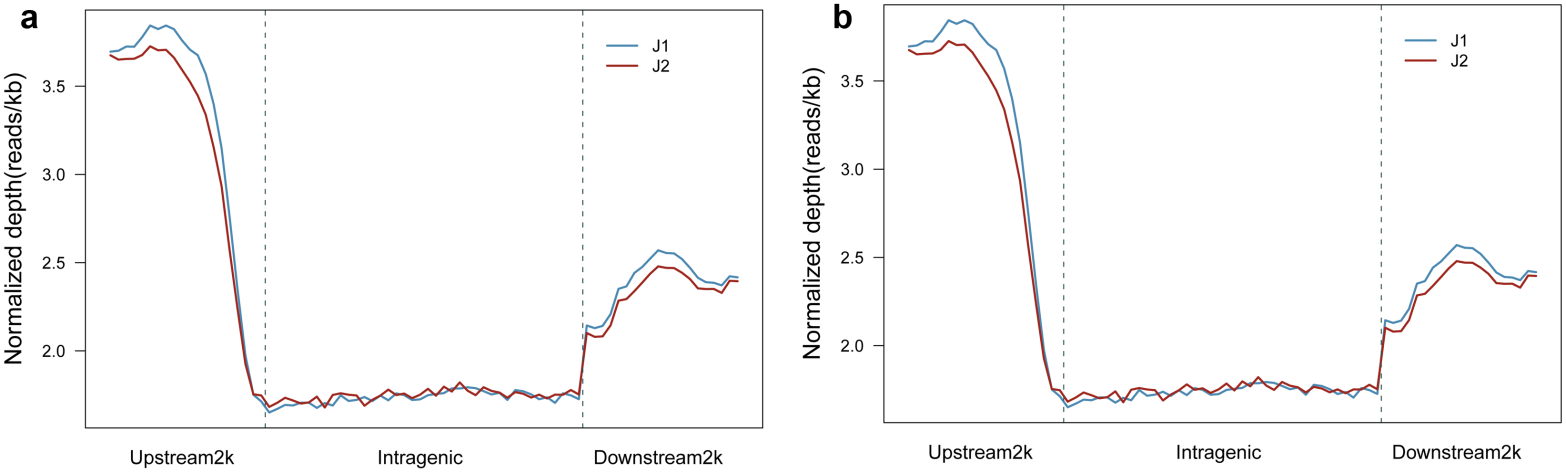
Distribution of reads around the gene body (a) and CpG islands (b). The x-axis indicates position around the gene body (a) and CpG island (b); the y-axis indicates the normalized read depth. The figure reflects methylation levels around the gene body and CpG islands.

We compared the DNA methylation levels between young (J1) and senescent cotyledons (J2) around the gene body. The DNA methylation level in both young (J1) and senescent cotyledons (J2) dramatically increased 2000 bp upstream of the transcription start site (TSS). Intragenic regions showed the lowest DNA methylation levels in both young (J1) and senescent cotyledons (J2). Moreover, we observed that regardless of cotyledon age, methylation levels increased after transcription terminal sites (TTSs) and then slightly decreased (Fig 3a). However, J1 cotyledons showed significantly higher DNA methylation levels in regions before TSSs and after TTSs than J2 (Fig 3a).

DNA methylation levels in both young (J1) and senescent (J2) cotyledons sharply increased at 2k upstream of CpG islands. Regardless of cotyledon age, the CpG islands demonstrated the highest methylation levels, which dramatically decreased the 2000 bp downstream of CpG islands (Fig 3b). However, the CpG islands in J1 showed higher methylation levels than those in J2.

### Distribution of highly methylated regions in J1 and J2

**Fig 4:**
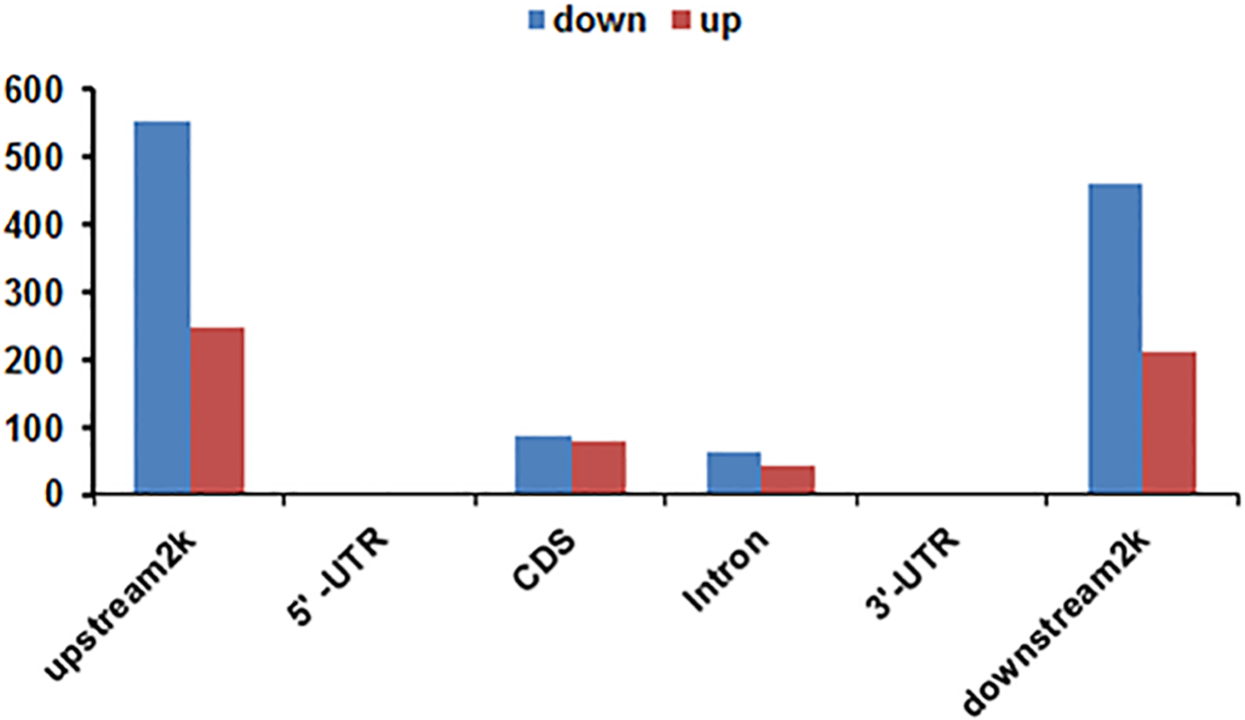
Number of genes in each specific gene element. The x-axis indicates different gene elements, and the y-axis indicates the different methylated gene numbers in each specific gene element. Different methylated gene elements were concentrated in the upstream 2k and downstream 2k.

There were 136,640 and 128,182 HMRs in J1 and J2, respectively. The mean HMR length was approximately 1400 bp, and the HMR coverage sizes were 8.97% of the genome in J1 and 8.53% in the J2 stage (Table 3). The chromosome location and enriched HMR tags in J1 and J2 are listed in S4 and S5 Tables, respectively. Considering the important role of CpG in DNA methylation, we calculated that 5–40 is the most abundant CpG number for HMRs in J1 and J2 (S6 Table). Analysis of HMR coverage for different genome components showed that genome coverage in coding sequences (CDS) accounted for a considerable proportion (S7 Table). Comparison of the gene methylation status showed that there were a total of 1940 different methylation regions (DMRs) (S8 Table), and comparison of J1 with J2 showed 625, 1, 80, 92, 2 and 422 genes down-methylated in the upstream 2k region, 5’- UTR, CDS, intron, 3’- UTR and downstream 2k region, respectively. When we compared the DMRs in J1 to J2, there were 278, 0, 81, 64, 0 and 200 genes up-methylated in the upstream 2k region, 5’- UTR, CDS, intron, 3’- UTR and downstream 2k region, respectively (Fig 4).

**Table 3:** Information for HMRs.

Sample	Total Peaks	Peak Mean Length	Peak Median Length	Peak Total Length	Peak Covered Size In Genome(%)
J1	136,640	1411.81	1239	192,910,390	8.97
J2	128,182	1430.95	1257	183,422,168	8.53

### Characterization of DNA methylation in CpG islands

**Fig 5:**
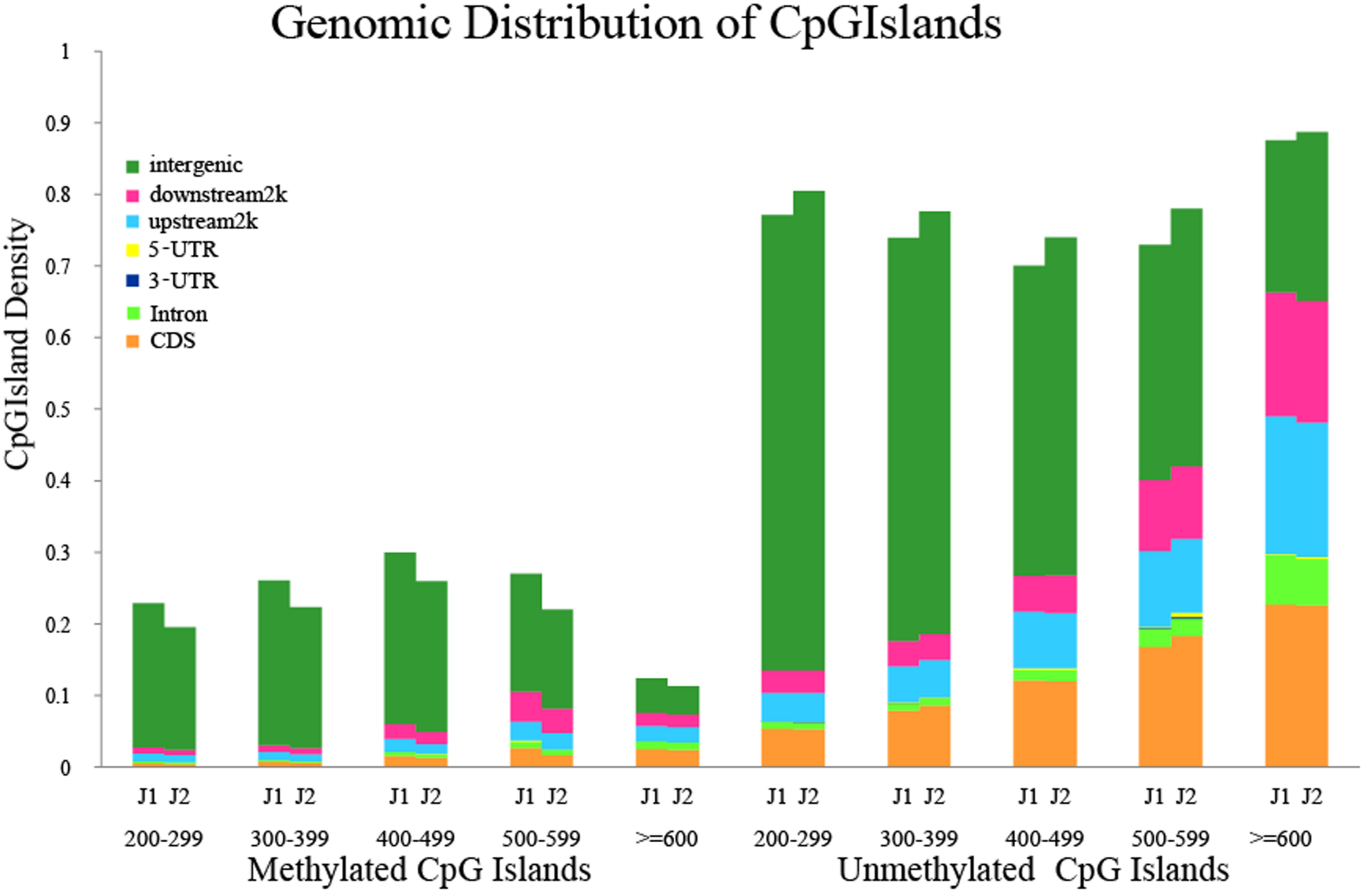
Genomic distribution of methylated and unmethylated CpG islands. We subdivided the CpG islands into methylated and unmethylated islands and categorized them into different bins according to their size. The number of CpG islands in a particular bin was calculated in different regions and was subsequently normalized to the total number of CpG islands in that bin.

Considering the large difference in reads distribution around CpG island regions between J1 and J2 (Fig 3b), we analyzed CpG islands in *G*. *hirsutum* L. and compared methylated CpG island distribution between J1 and J2. In total, 85,562 CpG islands were identified in the *G*. *hirsutum* L. genome (S9 Table). We performed BLAST analysis of the MeDIP-seq reads against the genome CpG islands and identified 65,962 and 65,752 methylated CpG islands in J1 and J2, respectively. We then calculated the MeDIP-seq reads mapped percentage and depth for each CpG island (S10 Table). To further analyze the methylation status of CpG islands between young (J1) and senescent cotyledons (J2), we classified CpG islands into two classes, methylated CpG islands and unmethylated CpG islands. We considered CpG islands containing methylated peaks to be methylated, and the rest were considered unmethylated. Most of the methylated CpG islands were located within intergenic regions. Within the gene body, CDSs contained more methylated CpG islands than UTRs and introns. Moreover, when we classified methylated CpG islands according to their sizes, there were more methylated CpG islands in the 200- to 299-bp range (Fig 5). There were more unmethylated CpG islands than methylated CpG islands in each size. The CpG island size and density increased in the CDS, upstream 2k and downstream2k.

### MeDIP-seq data validation by bisulfite sequencing

Bisulfite sequence PCR was performed to validate the MeDIP-Seq results from young (J1) and senescent cotyledons (J2) (S11 Table). Randomly selected two down-methylated genes (*CotAD_14795* and *CotAD_48340*) upstream 2k and three up-methylated genes (*CotAD_27532*, *CotAD_44113*, and *CotAD_39214*) up-stream 2k and a senescence-associated gene (*CotAD_20715*, *GhSAG101*) were used to check DNA methylation patterns with the bisulfite sequence method. The methylation patterns of all five genes were the same as the DNA methylation patterns obtained with the MeDIP-seq data, indicating that the MeDIP-seq method is a reliable technique to compare methylation levels between young (J1) and senescent (J2) cotyledons.

### GO analysis

**Fig 6:**
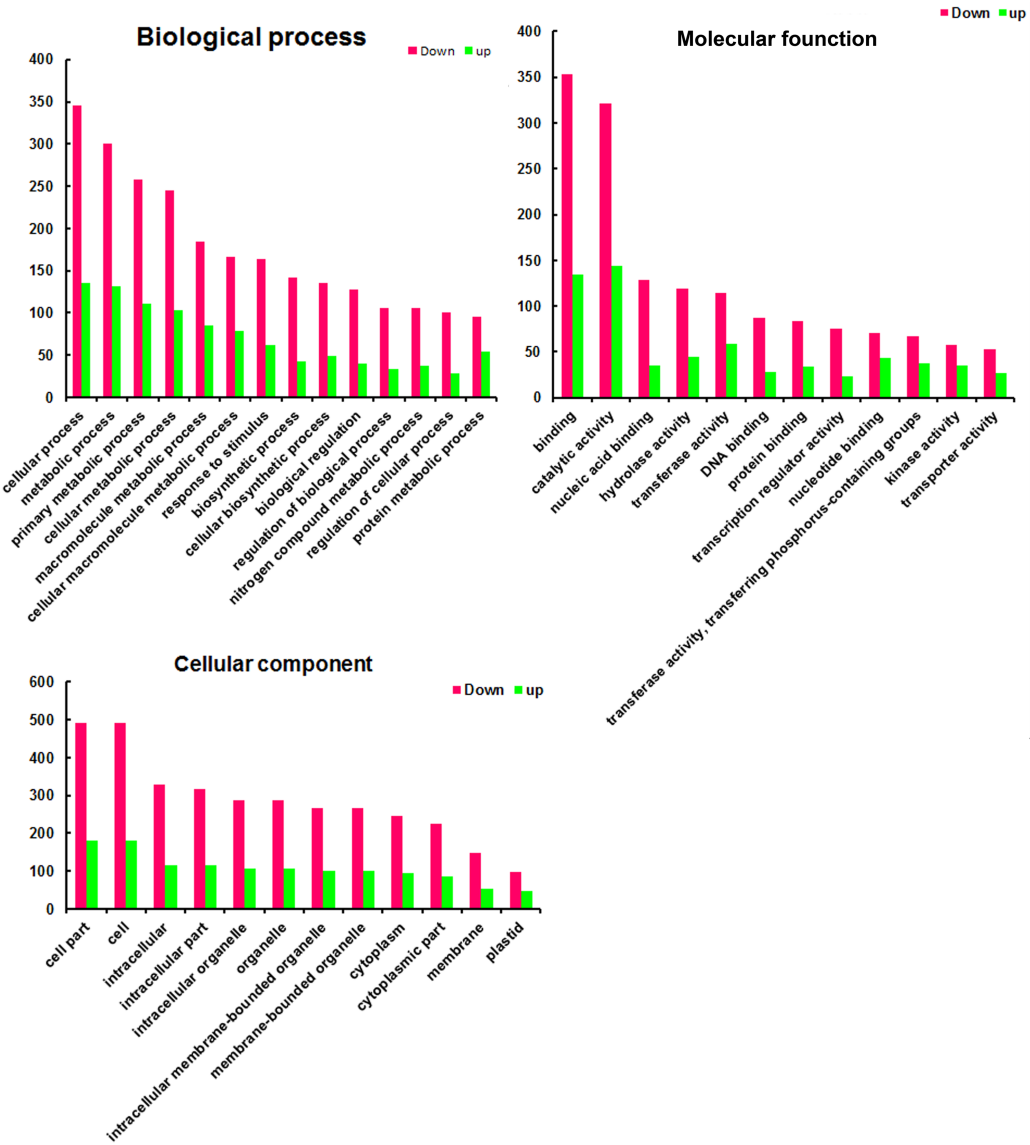
GO category analysis of down-methylated and up-methylated genes at J1 and J2 cotyledon stages. The green indicates down-methylated genes, and the red indicates up-methylated genes, which were defined with the FDR <0.05 and at least a 2.0-fold-change in read number. The results indicate GO annotations with p values less than 0.05.

According to the agriGO analysis of methylated genes against the *Gossypium* background GO annotation data could be clustered into three categories: biological process, cellular component and molecular function. There were 5115 down-methylated genes and 1541 up-methylated genes in the cellular component data, primarily focused on cell part, cell, intracellular, intracellular part and intracellular organelle; 3648 down-methylated genes and 765 up-methylated genes in molecular function, primarily focused on binding, catalytic activity, nucleic acid binding, and hydrolase activity; and 8274 down-methylated genes and 1739 up-methylated genes in biological process, primarily enriched for cellular process, metabolic process, primary metabolic process, cellular metabolic process, macromolecule metabolic process, cellular macromolecule metabolic process (Fig 6). Detailed information is provided in S14 Table.

### Methylated genes are significantly enriched for the biochemical pathway

DNA methylation in the CDS, downstream 2k, intron, and upstream 2k usually has different effects on gene expression. Therefore, we analyzed the biochemical pathways of all gene elements. We used the KAAS (KEGG Automatic Annotation Server) and pathway enrichment analysis tool of OmicShare software for all DMR-related gene elements to determine the functional pathways involved. According to the pathway enrichment analysis there were only 2 significantly enriched pathways in the downstream 2k and 6 significantly enriched pathways in the upstream 2k, and most of the pathways belonged to carbohydrate metabolism, biosynthesis of other secondary metabolites, amino acid metabolism, signal transduction, lipid metabolism and metabolism of terpenoids and polyketides pathways (Table 4). These data suggest that DNA methylation plays an important role in regulating secondary metabolites in cotyledon senescence.

**Table 4:** Significantly enriched pathways in different gene elements.

Gene elements	Pathway	P-value	Q-value	Pathway ID
downstream2k	Plant hormone signal transduction	0.004571	0.0402011	ko04075
downstream2k	Arachidonic acid metabolism	0.0013919	0.04593376	ko00590
upstream2k	Pentose and glucuronate interconversions	0.005209	0.03901366	ko00040
upstream2k	Glycine, serine and threonine metabolism	0.0032251	0.0322513	ko00260
upstream2k	Starch and sucrose metabolism	0.0027532	0.0322513	ko00500
upstream2k	Limonene and pinene degradation	0.0016161	0.03636263	ko00903
upstream2k	Flavonoid biosynthesis	0.003721	0.01275	ko00941
upstream2k	Stilbenoid, diarylheptanoid and gingerol biosynthesis	0.0013143	0.03636263	ko00945

### DNA methylation-associated gene expression analysis

There are two mechanisms influencing the methylation of cytosine: DNA methylation and DNA demethylation. DNA methyltransferase genes such as *GhCMT3*, *GhDRM1/2*, *GhDRM3*, *GhMET1* and *GhDDM1* function in the maintenance or de novo generation of DNA 5-methylcytosine. *GhNERD*, *GhNRPD*, *GhSAHH1a*, and *GhSAHH1b* encode proteins that are also required for DNA methylation. However, DNA demethyltransferases such as ROS1 and DME remove methyl groups from 5-methylcytosine In the *G*. *hirsutum* L. genome sequence, we identified a total of 13 DNA methyltransferase genes and 4 DNA demethyltransferase genes (S12 Table).

**Fig 7:**
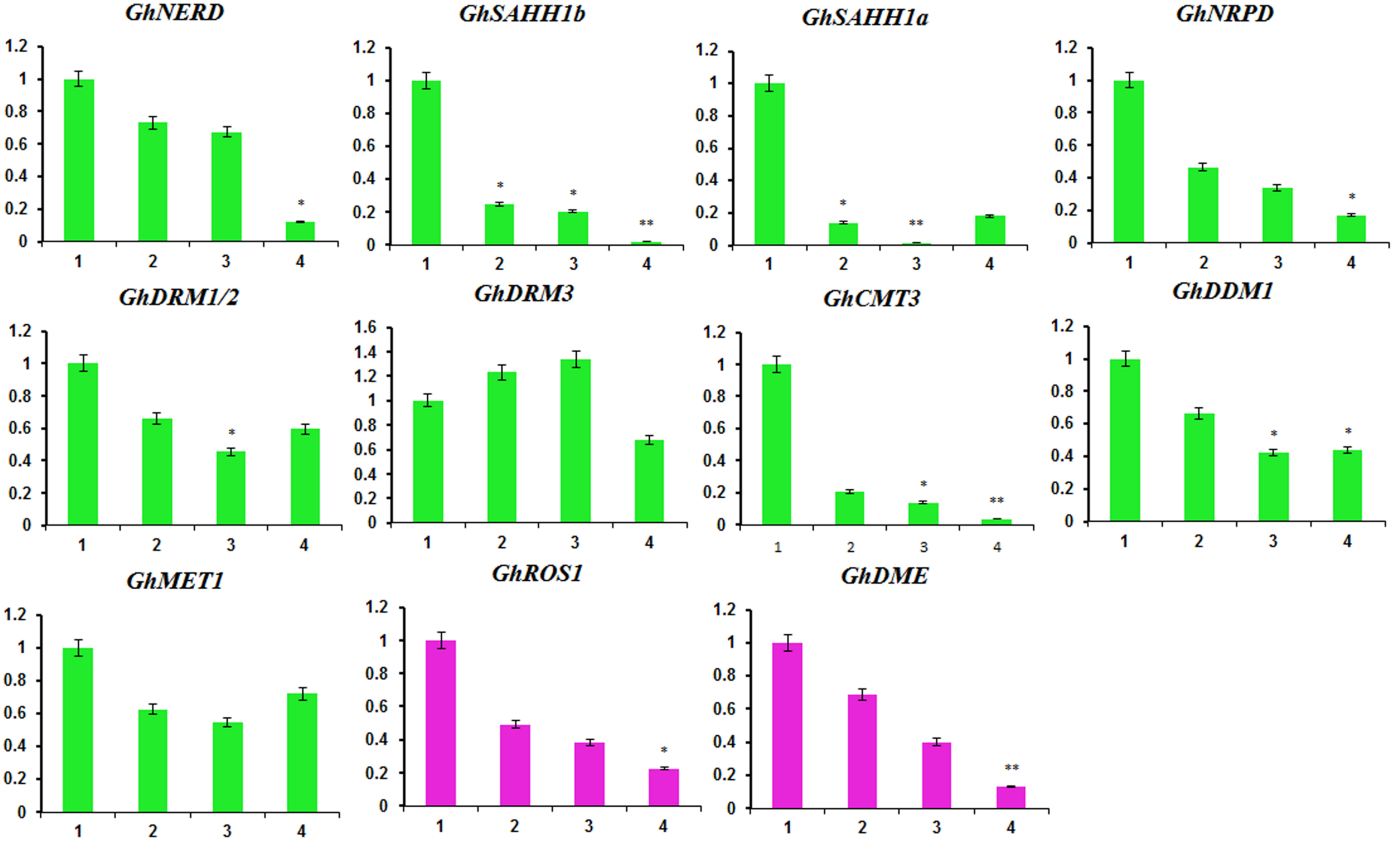
Relative expression of DNA methylation-associated genes at the four developmental stages. In the X-axis, 1, 2, 3 and 4 indicate the four cotyledon development stages from young to senescent. The y-axis indicates relative expression values of qRT-PCR compared with the first stage. The bars show the standard deviation of three technical replications. * indicates significant difference at *P*<0.05 level, ** indicates extremely significant difference at *P*<0.01 level.

Is the mechanism causing DNA methylation levels to decrease from young to senescent cotyledons due to DNA methylation or DNA demethylation? DNA methyltransferase-associated genes and DNA demethyltransferase-associated genes were selected for assessment by qRT-PCR in young and senescent cotyledons. According to the qRT-PCR results, both DNA methylation-associated and DNA demethylation-associated genes showed decreased expression values with increased senescence (Fig 7), suggesting that senescent cotyledons exhibit lower DNA methylation levels than young cotyledons due to decreased expression of methyltransferase-associated genes.

## Discussion

### DNA methylation is widely involved in *G*. *hirsutum* L. cotyledon development

Leaf development from youth to senescence involves changes to gene expression, protein translation and modification, cellular structure, metabolic pathways, and plant hormone levels, among others. In recent years, DNA methylation has been reported as an important epigenetic modification regulating the leaf senescence process [25].

In this study, we used MeDIP-Seq methods to sequence young (J1) and senescent (J2) cotyledons in cotton. We produced 4G reads from both J1 and J2. These reads were widely spread on each chromosome (Fig 2). The read distribution along a gene indicated that young cotyledons exhibit more DNA methylation in both 2k upstream and 2k downstream sites than senescent cotyledons, and the read distribution around CpG islands indicated that young cotyledons show higher DNA methylation levels in CpG islands than senescent cotyledons. By comparing methylated gene components 2k upstream, CDS, introns, and 2k downstream, we identified 1940 DMR-related genes between young (J1) and senescent (J2) cotton cotyledons. According to the results, senescent leaves (J2) have lower DNA methylation levels than young cotton leaves (J1). With the same sequence depth, the CpG percentage and CHH were higher in J1 than J2. There were more HMRs overlapping with genetic elements in J1 than in J2, suggesting that there were more methylated DNA regions on gene elements in young (J1) cotyledons. In young (J1) cotyledons, methylation was higher around the gene body and CpG islands than in senescent cotyledons (J2). In J1, the upstream 2k region showed higher DNA methylation levels than J2, and CpG islands also showed higher DNA methylation levels than J2, which usually occurs at promoter regions to control gene expression [26].

Read distribution around gene body and CpG islands revealed that young cotyledons (J1) show higher DNA methylation levels at regions 2k upstream, 2k downstream and at CpG islands than senescent cotyledons (J2). According to HMR-related genes analysis, as plants progress from young to senescent cotyledons, there are more HMR-covered genes down-methylated, indicating that as cotyledons age, DNA methylation levels decrease in cotton, which is consistent with previous studies in *P*. *radiata* [11] and *Q*. *robur* seeds [15]. In *Arabidopsis*, several senescence-associated genes (SAGs) are regulated by DNA methylation. *AtSAG24*(At1G66580), *AtSAG113*(At5G59220), *AtSAG14*(At5G20230), *AtSAG1*(At2G43820), *AtSAG20*(AT3G10985), *AtSAG102*(AT3G63210), *AtSAG15*(AT5G51070), *AtSAG101*(AT5G14930), *AtSAG13*(AT2G29350), and *AtSAG18*(AT1G71190) all have methylated cytosines that were identified with a single-base resolution method [27]. In cotton, we used bisulfite sequencing PCR and found that *GhSAG101* (CotAD_20715) can be methylated on gene body with the DNA methylation level decreased from J1 to J2 (S13 Table).

From young to senescent, the metabolic shift is important for nutrient mobilization, and some studies have demonstrated that secondary metabolites could reflect the leaf senescence process. Leaf senescence increased, the ratio of Gly/Ser was high with low photosynthesis activity; and plant senescence usually accompanied proteolysis [28]. In leaves, starch and sucrose metabolism could affect the level of sugar; however, their role in leaf senescence is still controversial [28]. At the cellular level, leaf senescence is accompanied by chloroplast degradation, decreased chlorophyll content and arginine levels, free amino acid accumulation, membrane lipid peroxidation, increased putrescine acid content, and enzyme activity, etc. In our work, GO and nr annotation analysis revealed that differentially methylated genes between young (J1) and senescent cotyledons (J2) broadly participate in cellular components, biological processes and molecular functions (Fig 6). These differentially methylated genes were significantly enriched in several biochemical pathways, especially secondary metabolite processes (Table 4). There are some studies about the epigenetic regulation of secondary metabolite biosynthesis in fungi but few studies about the epigenetic regulation on plant secondary metabolites. According to our results, we can conclude that DNA methylation is a very important epigenetic modification that is highly regulated during cotyledon development.

In this study, we used MeDIP-seq to comprehensively analyze DNA methylation in young (J1) and senescent (J2) cotyledons. Above all, DNA methylation is one of the most important epigenetic modifications in regulating the plant senescence process. DNA methylation levels decreased during the cotton cotyledon senescence process, which affected the expression of many genes and some biochemical pathways involved, particularly those regulating secondary metabolite processes (Table 4).

### DNA methylation of repeat elements and CpG islands involved in *G*. *hirsutum* L. cotyledon senescence

Repeat elements showed very high DNA methylation proportions in both J1 and J2 which indicated that DNA methylation in repeat elements is very important in *G*. *hirsutum* L. *G*. *hirsutum* L. is allotetraploid cotton with a large genome size; repeat elements make up 66% of the *G*. *hirsutum* L. genome, and LTR/Gypsy and LTR/Copia are the most predominant repeat elements [24]. In this work, repeat elements exhibited a larger proportion of methylated elements than genes, which agrees with previous work of DNA methylation analysis in cotton fiber development and DNA methylation of horse MeDIP-seq [22,29]. In some other plant species, gene elements make up a larger proportion than repeat elements, such as DNA methylation which occurs frequently in *Arabidopsis*, and methylcytosine, which occurs in repeat elements to help protect genomic structure and affect gene expression [30]. Loss of DNA methylation to SINEs in *A*. *thaliana* ectopically activates expression of FWA (FLOWERING WAGENINGEN) and results in late flowering [31]. DNA methylation patterns are different in different tissues and different developmental stages [32].

Compared with *T*. *cacao* and *A*. *thaliana*, more repeat elements were inserted near (within 1 kb) genes in cotton [33], and repeat elements were more likely to be methylated [34]. CpG islands are usually located near gene promoters and can also appear within or at the 3’- end of genes in the human genome [5]. Promoters containing repeat elements exhibited higher DNA methylation level than gene, 5’- UTR, CDS and 3’- UTR in *A*.*thaliana* [35]. Read distribution around CpG islands showed that CpG islands have higher DNA methylation than the up- or downstream 2k. Comparing repeat elements and CpG islands to the gene body, it seems reasonable that the upstream and downstream 2k show higher methylation levels than gene body based on the DNA methylated read distribution around the gene body.

DNA methylation modified repeat elements and CpG islands, which could regulate gene expression to regulate cotton cotyledon senescence. Also, it has reported that DNA methylation could mediate gene and repeat element expression to enhance the transition from epidermal to fiber cells during ovule and seed development in cotton [34].

### Senescent cotton cotyledons (J2) show lower DNA methylation levels than young cotyledons (J1)

DNA methylation is a highly regulated process. In plants, three DNA methylation mechanisms, DNA methylation maintenance, de novo DNA methylation and DNA demethylation balance DNA methylation levels. There are three types of cytosines that can be methylated, CHH, CHG and CG. In *Arabidopsis*, CHROMOMETHYLASE 3 (CMT3) is a plant-specific DNA MTase that maintains CHG and CHH DNA methylation [36]. DNA METHYLTRANSFERASE 1 (MET1) functions in the maintenance and DNA methylation of CG sites [37]. In higher plants, domains rearranged methylase 1/2 (DRM1/2) shows de novo methylation activity in all sequence contexts and acts redundantly with CMT3 to maintain methylcytosine [38]. DRM3 functions in the maintenance of non-CG DNA methylation and the establishment of RNA-directed DNA methylation triggered by repeat sequences and the accumulation of repeat-associated small RNAs [39]. DNA methyltransferases place a methyl-group on the cytosine of DNA, during which the S-adenosylhomocysteine hydrolase1 (SAHH1) provides the substrate. DNA methylation also requires the NEED FOR RDR2-INDEPENDENT DNA METHYLATION (NERD) and NUCLEAR RNA POLYMERASE D1B (NRPD1B) genes, which play critical roles in methyl homeostasis [40]. In *A*.*thaliana*, the DNA glycosylase gene family, which consists of DME, ROS1 (also known as DML1), DML2 and DML3, regulates DNA demethylation by removing methyl groups from 5-methylcytosine [41].

During the leaf senescence process, chlorophyll, protein, nucleic acids and some macromolecules are degraded and the leaf becomes yellow, which is the recession and death process [42]. In Fig 1, we observed that the color of J1 is a healthy green; however, the margin is yellow and the center is yellowish-green in J2. With the growing days and phenotypes of J1 and J2, we judged that J2 cotyledons represent senescence. According to qRT-PCR analysis, both DNA methylation-associated and DNA demethylation-associated genes showed relatively decreased expression levels (Fig 7) from young to senescent cotyledons, which might explain the DNA methylation level decrease in young to senescent cotyledons because DNA methylation associated genes decrease in expression with the aging process.

## Conclusion

In this study, we compared global DNA methylation between young (J1) and senescent (J2) cotyledons in *G*. *hirsutum* L. by MeDIP-Seq. We concluded that young cotyledons have higher DNA methylation levels than senescent cotyledons. The methylated sequences were broadly distributed across all 26 chromosomes, and DNA methylation-associated genes were significantly involved in secondary metabolites. We investigated the expression patterns of 9 DNA methyltransferase-associated genes and 2 DNA demethyltransferase-associated genes from young to senescent cotyledons and found that they were down-regulated during senescence, suggesting that senescent cotyledons have lower DNA methylation levels because of decreased DNA methylation activity. This work comprehensively compared global DNA methylation levels between young and senescent cotyledons. Considering the shortcomings of MeDIP, a single-base resolution DNA methylation map should be defined in the future.

## Methods

### Plant materials

In this work, we used cotyledons of Liao4086 to perform a global analysis of DNA methylation in young and senescent *Gossypium hirsutum* L. by MeDIP-Seq, because cotyledons grow quickly and could be well controlled without being damaged by disease and insects. Healthy and uniform Liao4086 cotton seeds were grown in a greenhouse at the Cotton Research Institute of Chinese Academy of Agricultural Sciences, Anyang, Henan Province. Under 30°C light/22°C dark and 16 h light/8 h dark conditions, the seeds were strictly germinated at the same depth of soil to ensure that the cotton seeds broke through the soil at the same time [43,44]. The materials were collected in triplicate every two weeks from the first stage when the cotyledons flattened, and four time-points were collected in total. The first and fourth samples were used as the young cotyledon (J1) and senescent samples (J2), respectively. Chlorophyll color changed from green to yellow during the aging process (Fig 1).

### DNA extraction and preparation for MeDIP-seq

Genomic DNA was extracted from J1 and J2 samples using cetyl trimethylammonium bromide (CTAB) method [45]. Genomic DNA was treated with the following steps: sonication to generate DNA fragments of 300–500 bp, DNA-end repair, 3’-dA overhang, ligation of sequencing adaptors, denaturation of double-stranded DNA, immunoprecipitation by 5-mC antibody, real-time PCR validation, PCR amplification and size selection (usually 200–300 bp). Insert size was strictly controlled to be approximately 250 bp, and all of these processes were qualified with an Agilent 2100 Bioanalyzer and agarose gel electrophoresis [4]. Following PCR validation, DNA libraries were sequenced on an Illumina HiSeq 2000 (Illumina, CA, USA) to generate paired-end 49-bp reads by the Beijing Genomics Institute (BGI, China). The MeDIP-Seq data from this study was submitted to the NCBI Sequence Read Archive (SRA), and the accession number of J1 and J2 is SRP066408.

### Bioinformatics analysis

The raw reads obtained from Illumina sequencing were filtered to remove adaptor sequences, sequences containing N more than 10% reads and low-quality reads [46]. The clean data were stored in fastq format. The filtered reads were mapped to the *G*. *hirsutum* L. reference genome [24] with SOAPaligner v2.21, and reads with no more than 2 mismatches were considered for further analysis [47]. The uniquely mapped reads were used to analyze reads distribution in the *G*. *hirsutum* L. genome and the distributions of different components.

To evaluate our MeDIP-Seq data, we analyzed cytosine base (C) coverage. The methylated cytosine bases in the eukaryote genome are generally one of three forms: CG, CHG or CHH (H indicates A, Tor C). Therefore, it is necessary to analyze genome-wide coverage of the three forms at cytosine sites using different sequencing depths on the Crick strand, the Watson strand, and both strands to evaluate the sequence strategy. Because of the high DNA methylation frequency, we analyzed CpG density in specific regions. We divided the genome into 1000 bp windows and calculated the distribution of CpG density. To characterize MeDIP-seq reads around the gene body and CpG islands, we divided both the upstream and downstream 2 kb of the gene body into 20 equal regions each to generate 40 equal regions. We calculated the normalized number of reads for each region and used the same method of read distribution around the gene body to calculate the read distribution around CpG islands. Analysis of MeDIP-Seq data was conducted in the *R* environment by *MEDIPS* package [48] and.a flow chart of analysis process could be found in S15 Table.

CpG Island Searcher (http://cpgislands.usc.edu/) was used to identify CpG islands (CGIs) according to the following criteria: DNA sequence length of more than 200 bp, GC content ≥50%, CpG observed/expected (o/e) ratio ≥0.6 and the gap between adjacent islands should be more than 100 bp. CpG islands overlapping with the methylated peaks were considered methylated [49,50].

Uniquely mapped reads were used to analyze highly methylated regions (HMRs), also known as peaks, based on a defined analysis model from MACS 1.4.0 software, and peaks with a p value less than 1e-5 were used for further analysis. The peaks of J1 and J2 were merged as candidate DMRs, and the number of normalized reads of each peak was tested by Chi-square with p-value <0.01. For each candidate, DMR was deemed differentially methylated between J1 and J2 with a false discovery rate (FDR) <0.01 and at least a 2.0-fold-change in read number [51]. DMR analysis was performed in R package (http://www.r-project.org). We calculated the HMR coverage by dividing the total length of regions in a specific element covered by HMRs by the total element length.

We defined methylated genes as HMRs that overlapped the gene element by more than 50% [8]. Methylated genes were used for GO annotation enrichment in biological process, cellular component and molecular function analysis using the online software agriGO (http://bioinfo.cau.edu.cn/agriGO/analysis.php), and the statistical test method of GO annotation was Fisher [52]. The KAAS online software (http://www.genome.jp/tools/kaas/) was used to identify the Ko number of methylated genes. Furthermore, according to the Ko numbers, pathway enrichment analysis was performed using OmicShare tools (http://www.omicshare.com/tools/Home/Soft/pathwaygsea), methylated genes were used to identify biochemical pathways significantly enriched in young (J1) and senescent cotyledons (J2), and p-values and corrected p-values (q-value) less than 0.05 were considered statistically significant [53].

### Bisulfite sequencing PCR analysis

Genomic DNA samples of J1 and J2 were extracted according to CTAB methods. The genomic DNA samples from J1 and J2 were treated with a DNA bisulfite conversion kit (Tiangen, China). MethPrimer (http://www.urogene.org/cgi-bin/methprimer/methprimer.cgi) was used to predict CpG islands. Primers were designed using MethPrimer and Oligo 7 software (S13 Table). MightyAmp DNA Polymerase Ver. 2 (TAKARA, Japan) was used to perform BS-PCR. PCR was performed in 50 μl reaction mixtures with 25 μl of MightyAmp Buffer Ver. 2, 1 μl of MightyAmp DNA Polymerase (1.25 U/μl), 2 μl of forward primers, 2 μl of reverse primers, 16 μl of ddH_2_O and 4 μl of modified DNA samples. The reaction was performed with the following program: 98°C for 2 min, followed by 40 cycles of denaturation at 98°C for 10 s, annealing at 60°C for 15 s and extension at 68°C for 40 s, and a final extension at 72°C for 10 min. The PCR product was purified using a TaKaRa Agarose Gel DNA Purification Kit, Ver. 2.0 (TAKARA, Japan). Purified DNA fragments were subcloned into the pMD18 T-vector (TAKARA, Japan), and 10 single clones were picked for each gene for sequencing (GENEWIZ, America) from J1 and J2 modified DNA samples. BIQ Analyzer software (http://biq-analyzer.bioinf.mpi-inf.mpg.de/) was used to measure the DNA methylation status of the selected genes with young and senescent cotyledons.

We randomly selected 5 genes with DNA methylation in their upstream 2k region and a senescence-associated gene (*CotAD_20715*, *GhSAG101*) (S10 Table) and used the MethPrimer program to predict CpG islands in the upstream 2k regions. We analyzed the sequenced data using BIQ Analyzer software (http://biq-analyzer.bioinf.mpi-inf.mpg.de/). For each sample, we analyzed methylation data by calculating the percentage of methylated CpGs from the total number of CpGs.

### RNA extraction and preparation for qRT-PCR

Triplicate cotyledon samples for each time-point were mixed in equal parts and used to extract total mRNA with a polysaccharides- and polyphenolics-rich RNAprep pure plant kit according to the manufacturer’s instructions (Tiangen, China). RNA integrity was measured with agarose gel electrophoresis, and the concentration was measured with a NanoDrop 2000/2000c (Thermo Scientific, USA). We downloaded DNA methyltransferase and demethyltransferase protein sequences of *A*. *thaliana*, which were used as query sequences for local BLASTp against the *G*.*hirsutum* L. genome protein sequence with 1e-30 to retrieve DNA methyltransferase and demethyltransferase genes in *G*.*hirsutum* L. [22, 34, 54]. A first-strand cDNA synthesis kit (TOYOBO, Japan) was used to synthesize cDNA. The PCR reaction contained 2 μg RNA, 4 μl 5×RT Master mix, and sterile ddH_2_O for a total volume of 20 μl. Synthesized cDNA samples were diluted 10-fold and then used for qRT-PCR. qPCR was performed on an ABI7500 system (Applied Biosystems, USA) with three technical repeats. For each 20 μl reaction, 10 μl 2×SYBR Green I Master Mix, 7.2 μl distilled H2O, 0.8 μl primers (final concentration 0.4 μM) and 2 μl cDNA templates were added. Amplification reactions were initiated with a pre-denaturing step (95°C, 10 min), followed by denaturing (95°C, 10 s), annealing (60°C, 30 s) and extension (72°C, 30 s) for 40 cycles [54]. Two reference genes were used to normalize the target genes, and the primers are listed in S16 Table. The relative gene expression level were calculated by the 2^-ΔΔCt^method [55].

## Supporting information

S1 Table Genome coverage of three types of methylated nucleotide sites (CG, CHG, and CHH) under different sequencing depths. (XLSX)

S2 Table Distribution of MeDIP-Seq reads in different CG density regions. (A): J1; (B): J2.
(XLSX)

S3 Table Genome coverage distribution across sequencing depth of J1 and J2. (XLSX)

S4 Table Genomic distribution of uniquely mapped reads. (XLSX)

S5 Table Highly methylated regions in J1. (XLSX)

S6 Table Highly methylated regions in J2. (XLSX)

S7 Table CpG numbers in HMRs. (XLSX)

S8 Table HMR genome coverage in different genome components. (XLSX)

S9 Table DMR-related gene information. (XLSX)

S10 Table CpG islands in *G*. *hirsutum* L. (XLSX)

S11 Table Methylated CpG Islands in J1 and J2. (XLSX)

S12 Table DNA methyltransferase and demethyltransferase genes of *A*. *thaliana* and *G*. *hirsutum* L. (XLSX)

S13 Table Validation of MeDIP-seq data by bisulfite sequencing. (DOCX)

S14 Table Detailed information of the cellular component, molecular function and biological function between down-methylated and up-methylated genes. (XLSX)

S15 Table A flow chart for MeDIP-Seq quality evaluation analysis by *MEDIPS* package in *R* environment. (DOCX)

S16 Table Primer sequences for bisulfite sequencing and qRT-PCR. (XLSX)
