# Supplementary material for: Global analysis of DNA methylation in young (J1) and senescent (J2) Gossypium hirsutum L. cotyledons by MeDIP-Seq
Source: PLoS One. 2017 Jul 17;12(7):e0179141. doi: 10.1371/journal.pone.0179141 (PMC5513416; doi:10.1371/journal.pone.0179141)
Supplement: S13 Table — (DOCX) [file pone.0179141.s013.docx]

**S13 Table. Validation of MeDIP-seq data by bisulfite sequencing.**

S13a. DNA methylation status of upstream 2kb of *CotAD_20715.*


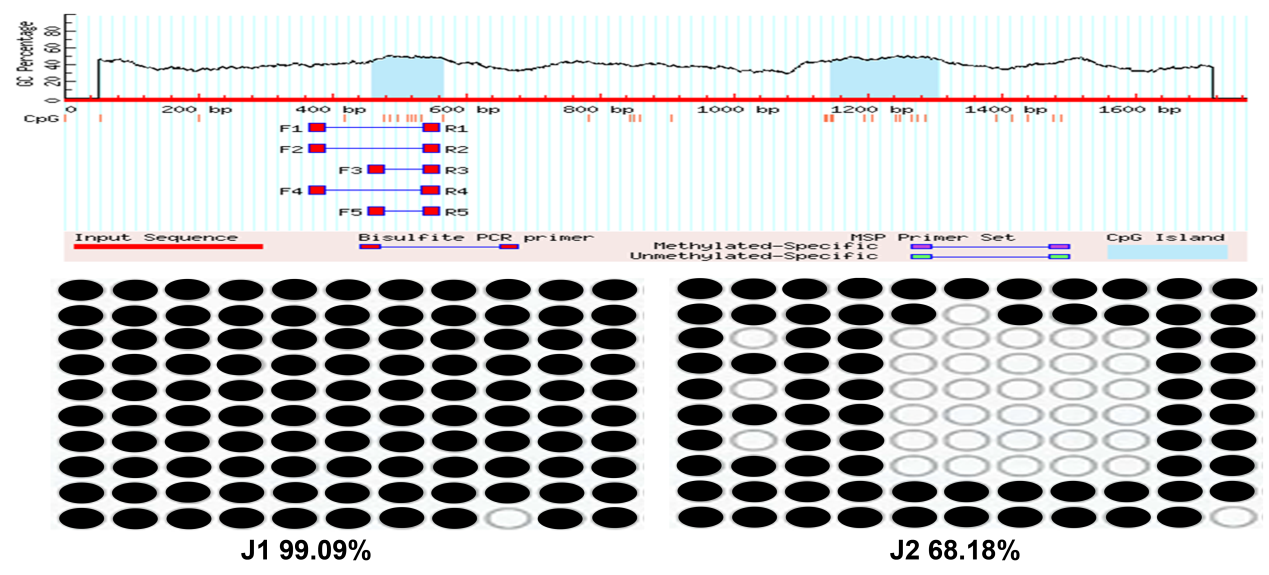


CpG islands of gene body of *CotAD_20715* were predicted by MethPrimer. F1-R1 regions were chosen as the analyzed regions. DNA methylation status of *CotAD_20715* was analyzed by bisulfite sequencing. 11 circles in each horizontal line represent 11 CpGs of *CotAD_20715*. Unfilled (white) and filled (black) circles represent unmethylated and methylated CpGs, respectively. Horizontal lines of circles represent one separate clone that was sequenced. The circos diagrams were generated by BIQ Analyzer software. For each sample, the methylation data were analyzed by computing the percentage of methylated CpGs of the total number of CpGs. J1 and J2 represent the young and senescence cotyledon stages.

**S13b. DNA methylation status of upstream 2kb of *CotAD_14795.***


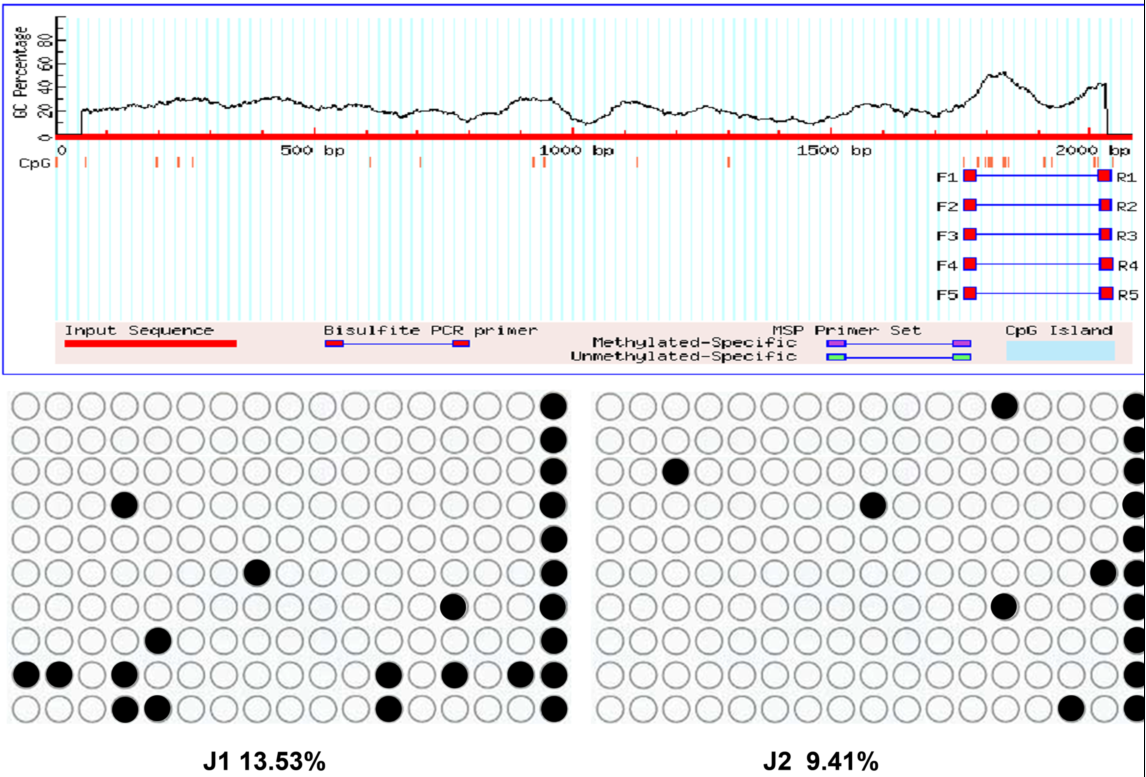


CpG islands of upstream 2k of *CotAD_14795* were predicted by MethPrimer. F1-R1 regions were chosen as the analyzed regions. DNA methylation status of *CotAD_14795* was analyzed by bisulfite sequencing. 17 circles in each horizontal line represent 17 CpGs of *CotAD_14795*. Unfilled (white) and filled (black) circles represent unmethylated and methylated CpGs, respectively. Horizontal lines of circles represent one separate clone that was sequenced. The circos diagrams were generated by BIQ Analyzer software. For each sample, the methylation data were analyzed by computing the percentage of methylated CpGs of the total number of CpGs. J1 and J2 represent the young and senescence cotyledon stages.

**S13c. DNA methylation status of upstream 2kb of *CotAD_39214.***


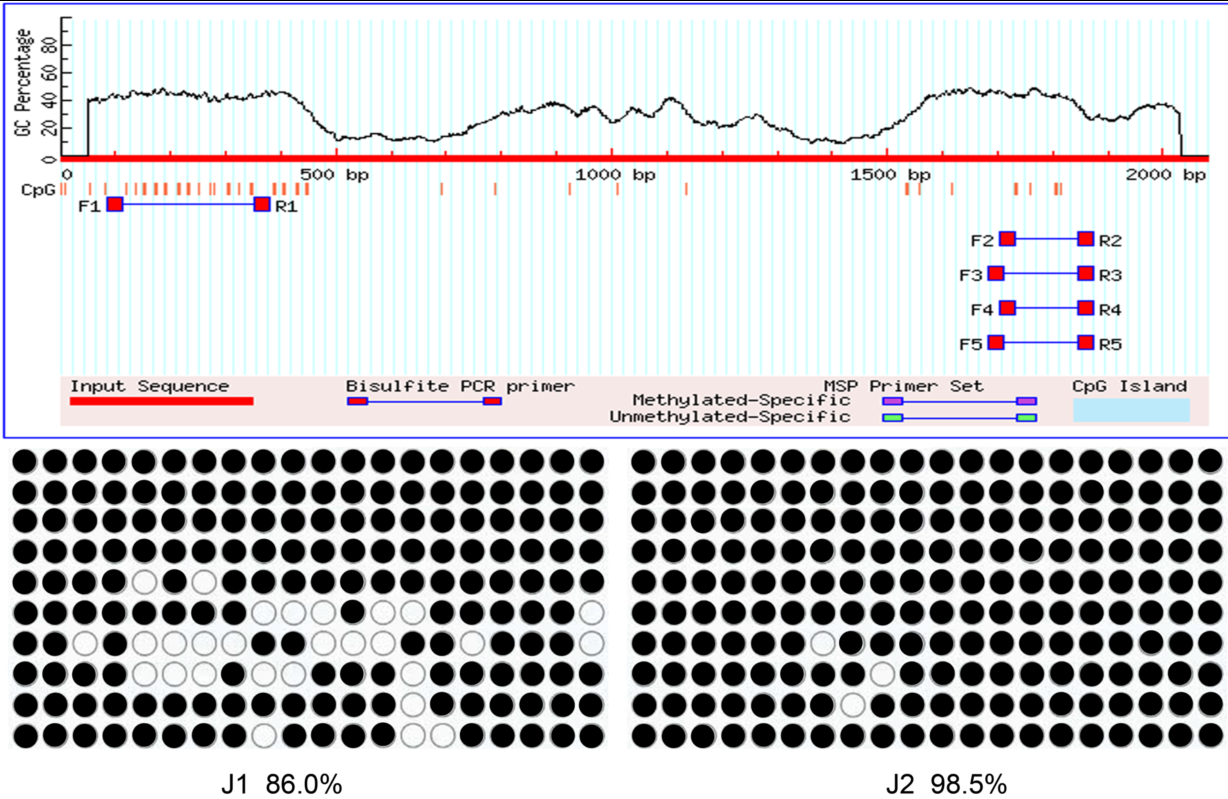


CpG islands of upstream 2k of *CotAD_39214* were predicted by MethPrimer. F1-R1 regions were chosen as the analyzed regions. DNA methylation status of *CotAD_39214* was analyzed by bisulfite sequencing. 20 circles in each horizontal line represent 20 CpGs of *CotAD_39214*. Unfilled (white) and filled (black) circles represent unmethylated and methylated CpGs, respectively. Horizontal lines of circles represent one separate clone that was sequenced. The circos diagrams were generated by BIQ Analyzer software. For each sample, the methylation data were analyzed by computing the percentage of methylated CpGs of the total number of CpGs. J1 and J2 represent the young and senescence cotyledon stages.

**S13d. DNA methylation status of upstream 2kb of *CotAD_48340.***


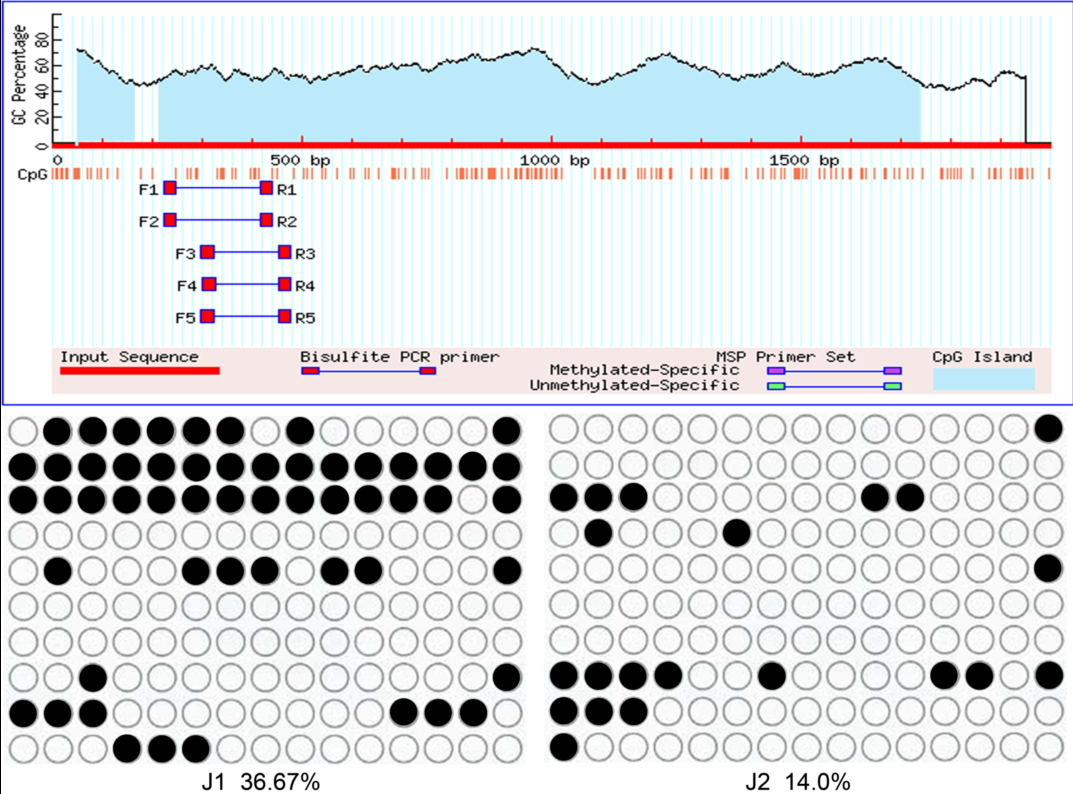


CpG islands of upstream 2k of *CotAD_48340* were predicted by MethPrimer. F1-R1 regions were chosen as the analyzed regions. DNA methylation status of *CotAD_48340* was analyzed by bisulfite sequencing. 15 circles in each horizontal line represent 15 CpGs of *CotAD_48340*. Unfilled (white) and filled (black) circles represent unmethylated and methylated CpGs, respectively. Horizontal lines of circles represent one separate clone that was sequenced. The circos diagrams were generated by BIQ Analyzer software. For each sample, the methylation data were analyzed by computing the percentage of methylated CpGs of the total number of CpGs. J1 and J2 represent the young and senescence cotyledon stages.

**S13e. DNA methylation status of upstream 2kb of *CotAD_27532.***


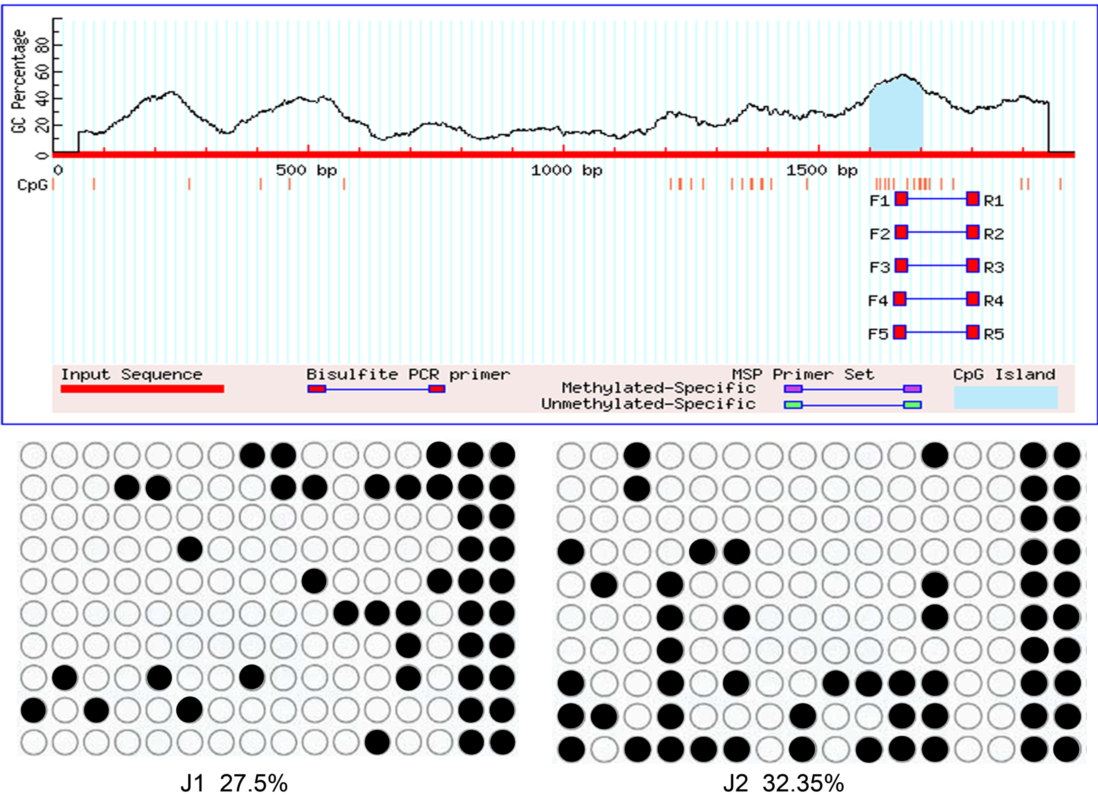


CpG islands of upstream 2k of *CotAD_27532* were predicted by MethPrimer. F1-R1 regions were chosen as the analyzed regions. DNA methylation status of *CotAD_27532* was analyzed by bisulfite sequencing. 16 circles in each horizontal line represent 16 CpGs of *CotAD_27532*. Unfilled (white) and filled (black) circles represent unmethylated and methylated CpGs, respectively. Horizontal lines of circles represent one separate clone that was sequenced. The circos diagrams were generated by BIQ Analyzer software. For each sample, the methylation data were analyzed by computing the percentage of methylated CpGs of the total number of CpGs. J1 and J2 represent the young and senescence cotyledon stages.

**S13f. DNA methylation status of upstream 2kb of *CotAD_44113.***


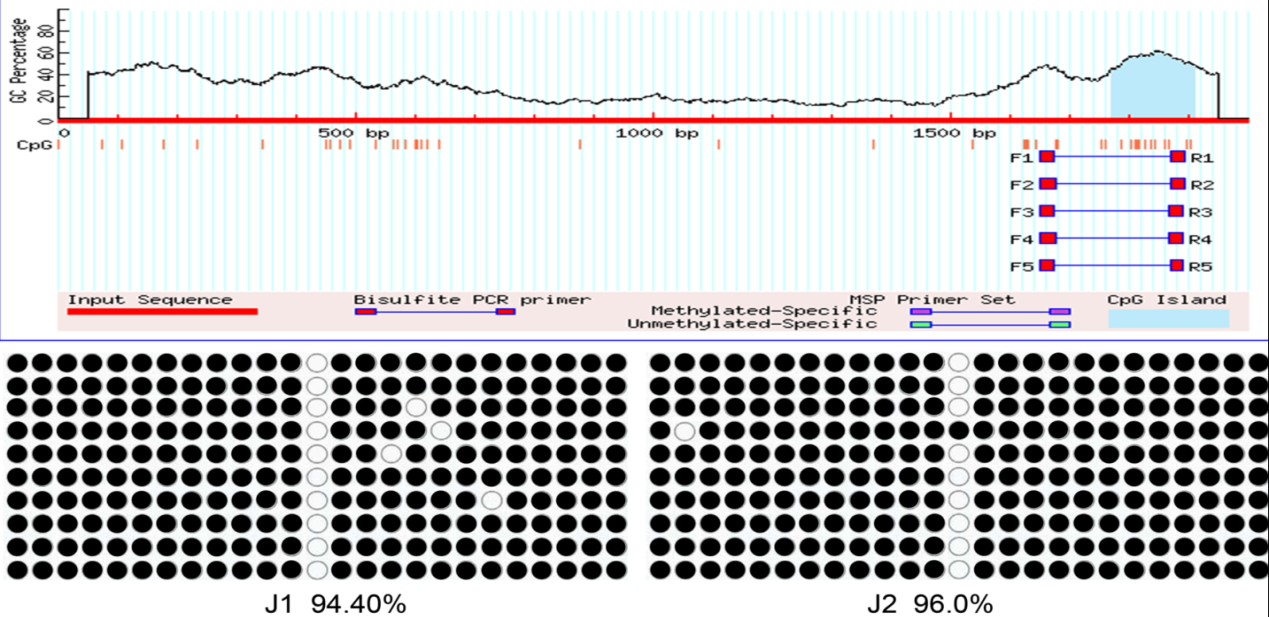


CpG islands of upstream 2k of *CotAD_44113* were predicted by MethPrimer. F1-R1 regions were chosen as the analyzed regions. DNA methylation status of *CotAD_44113* was analyzed by bisulfite sequencing. 25 circles in each horizontal line represent 25 CpGs of *CotAD_44113*. Unfilled (white) and filled (black) circles represent unmethylated and methylated CpGs, respectively. Horizontal lines of circles represent one separate clone that was sequenced. The circos diagrams were generated by BIQ Analyzer software. For each sample, the methylation data were analyzed by computing the percentage of methylated CpGs of the total number of CpGs. J1 and J2 represent the young and senescence cotyledon stages.
