# Supplementary material for: Global analysis of DNA methylation in young (J1) and senescent (J2) Gossypium hirsutum L. cotyledons by MeDIP-Seq
Source: PLoS One. 2017 Jul 17;12(7):e0179141. doi: 10.1371/journal.pone.0179141 (PMC5513416; doi:10.1371/journal.pone.0179141)
Supplement: S15 Table — (DOCX) [file pone.0179141.s015.docx]

**S15 Table**. A flow chart for MeDIP-Seq quality evaluation analysis by *MEDIPS* package in *R* environment.

MeDIP-Seq quality evalution

Coverage

(CHH/CHG/CG)

CG analysis

CpG islands

*MEDIPS.SeqCoverage*

*MEDIPS.exportWIG* and

*MEDIPS.CpGenrich*

*MEDIPS.createROIset*

Annotation

(Genomic features)

*MEDIPS.getAnnotation*

**part A**

**part B**

Install *R* environment and

*MEDIPS* package

**Note:** Part A indicates the flow chart for MeDIP-seq quality evalution and part B indicates documents of *MEDIPS* package in R environment. The scripts of all the documents are available online (http://www.bioconductor.org/packages/release/bioc/html/MEDIPS.html).
